# Clinicopathological features of recurrent papillary thyroid cancer

**DOI:** 10.1186/s13000-015-0346-5

**Published:** 2015-07-14

**Authors:** Jian Zhu, Xinli Wang, Xiaoxuan Zhang, Peifeng Li, Haifeng Hou

**Affiliations:** Department of Thyroid and Breast Surgery, General Hospital of Jinan Military Command, Jinan, 250031 China; Department of Pathology, Affiliated Hospital of Taishan Medical College, Taian, 271000 China; Medical Administration Division, General Hospital of Jinan Military Command, Jinan, 250031 China; Department of Pathology, General Hospital of Jinan Military Command, 25 Shifan Road, Tianqiao District Jinan, 250031 China; Department of Statistics, Taishan Medical College, Taian, 271000 China

**Keywords:** Clinicopathological features, Initial surgery approach, Papillary thyroid cancer, Pathological subtype, Recurrence

## Abstract

**Background:**

To investigate the clinicopathological features of recurrent papillary thyroid carcinoma (PTC).

**Methods:**

A retrospective analysis on clinical and pathological data of 34 patients with recurrent PTC was carried out. A total of 281 patients with non-recurrent PTC during the same time period were chosen as the control group.

**Results:**

Patients were divided into three groups according to the pathological subtype. The number of patients belonging to Groups 1, 2, and 3 were 28, 154, and 133, respectively. 78 patients underwent partial or whole thyroidectomy, 151 cases underwent thyroidectomy combining neck regional lymph node dissection, and 86 patients underwent thyroidectomy combining modified or radical neck dissection. Univariate analysis showed that PTC recurrence was associated with tumor size, extrathyroid invasion, initial surgery approach, lymph node metastasis, and pathological subtype (P < 0.05). Patient age, gender, complication with Hashimoto's thyroiditis, and multifocality were unrelated to PTC recurrence (P > 0.05). Multivariate analysis showed that initial surgery approach and pathological subtype perform important functions in PTC recurrence (P < 0.001). Initial surgery approach presented a negative correlation with PTC recurrence (β = −0.320, OR = 0.726). The pathological subtype was also related to PTC recurrence (β = 0.923, OR = 2.517).

**Conclusion:**

PTC patients without neck dissection showed greater likelihood of postoperative recurrence. Patients with the tall cell, columnar cell, diffuse sclerosing, and oncocytic variants showed a higher propensity for PTC recurrence after operation compared with those who did not. Tumor volume, extrathyroid invasion, and multiple lymph node metastases at the time of initial operation were also significantly related to postoperative recurrence. Follow-up supervision must be enhanced after initial treatment to mitigate PTC recurrence in susceptible patients. Effective and standard treatments must be adopted immediately after the discovery of recurrence.

## Background

Papillary thyroid carcinoma (PTC) is the most common thyroid cancer; this cancer presents relatively low malignancy, good prognosis, and a 10-year survival rate of over 90 %. However, the clinical behaviors of this cancer are complex and varied. PTC is easy to spread via lymphatic ducts, which results in recurrence, metastases, and even death [1]. Recurrent PTC mainly refers to localized and distant recurrence, including recurrence of the primary tumor, lymph node metastases, invasion of the esophagus and trachea, invasion of muscles, soft tissues and nerves, and distant metastases. Many factors can affect the recurrence of thyroid cancer, and final conclusion has not yet been reached. Many researchers believe that pathological type, staging, degree of extrathyroid invasion, lymph node metastatic rate, age, and initial surgery approach may be related to thyroid cancer recurrence. This research retrospectively analyzes the clinical data of 34 patients with recurrent PTC and presents a statistical comparison with non-recurrent patients to analyze factors related to PTC recurrence. The aim of the present work is to provide clinical evidence for standardized and appropriate treatment of recurrent PTC.

## Methods

### Clinical data

A total of 315 patients with PTC who were admitted to Jinan Military General Hospital from January 2009 to July 2013, including 66 male patients and 249 female patients, were recruited. The male to female ratio was 1:3.8. Ages ranged from 20 years to 78 years, and the median age was 48 years. The diameter of most tumors was less than 2 cm, the proportion of which was 47.1 % in the recurrent group and 85.8 % in the non-recurrent group. Moreover, 10.5 % (33 cases) of the patients presented extrathyroid invasion, and 42.2 % (133 cases) of the patients were found with lymph node metastases at the time of initial surgery. The tumors of 44.4 % (140 cases) of the patients showed multifocality, and 25.1 % (79 cases) of the patients had Hashimoto's thyroiditis (HT) (Table 1). This study received the approval of the ethics committee of the General Hospital of Jinan Military Command (No. 2013ZD01).

**Table 1:** Clinicopathological features of patients with recurrent and non-recurrent PTC

Clinicopathological feature	Recurrent group	Non-recurrent group	*χ* ^2^	P
	(*N* = 34)	(*N* = 281)		
Age/year			0.32	0.574
<45	14	130		
≥45	20	151		
Gender			3.11	0.078
Male	11	55		
Female	23	226		
Tumor size			34.76	<0.001
<1	3	130		
≥1- < 2	13	111		
≥2	18	40		
Initial surgery approach			11.39	0.003
First type	16	62		
Second type	14	137		
Third type	4	82		
Extra thyroidal invasion			22.15	<0.001
Yes	12	21		
No	22	260		
Coexistent Hashimoto’s thyroiditis			0.38	0.537
Yes	10	69		
No	24	212		
Lymphatic metastasis			10.10	0.001
Yes	23	110		
No	11	171		
Multifocality			0.00	0.968
Yes	15	125		
No	19	156		
Pathological subtype			34.45	<0.001
Group 1	11	17		
Group 2	20	134		
Group 3	3	130		

### Pathological subtype of PTC

Recruited PTC patients were divided into three groups. Patients with the tall cell, columnar cell, diffuse sclerosing, and oncocytic variants with higher invasiveness, higher probability of recurrence, and metastases were classified into Group 1. Patients with the follicular, clear cell, and conventional papillary carcinoma variants with similar prognosis were classified into Group 2. Patients with papillary microcarcinoma were classified into Group 3.

### Initial surgery approach

The methods of primary lesion dissection used in this research included thyroid lobectomy with or without isthmectomy and total/near-total thyroidectomy. The methods of lymph node dissection included central compartment node dissection, selective neck dissection, modified neck dissection, and radical neck dissection. During statistical analysis, thyroid lobectomy with or without isthmectomy and total/near-total thyroidectomy were classified as the first type of surgical approach. Surgical approaches belonging to the first type combined with central compartment node dissection, selective neck dissection, and wide dissection of metastatic lesions were classified as the second type of surgical approach. Finally, approaches combining modified neck dissection and radical dissection were classified as the third type of surgical approach.

### Statistical analysis

SPSS 17.0 software was used for statistical analysis. In addition, *χ*^2^-test and multivariate analysis using Cox proportional hazards modeling were carried out. Factors that may be related to PTC recurrence, including gender, age, tumor size, initial surgery approach, lymph node metastasis, number of lesions, complication with HT, extrathyroid invasion, and pathological subtype, were evaluated. *P* < 0.05 was considered statistically significant difference.

## Results

### Clinicopathological information

The clinicopathological information of PTC patients is given in Table 1, and the comparisons between patients with recurrence and non-recurrence were carried out. 34 recurrent patients (11 males and 23 females) were investigated in this study. The male to female ratio was 1:2.1. Ages ranged from 24 years to 72 years old, and the median age was 46 years old. Postoperative recurrence intervals from initial surgery ranged from five months to 18 years, with a median of 46 months. Twelve patients (35.3 %) showed postoperative recurrence within two years, 14 patients (41.2 %) showed postoperative recurrence within two to five years, and eight patients (23.5 %) showed postoperative recurrence over five years later. Recurrence occurred once in 29 patients (85.3 %), twice in 4 patients (11.8 %), and thrice in 1 patient (2.9 %). Two hundred eighty-one non-recurrent PTC patients were classified into the control group (55 males and 226 females); in this group, the male to female ratio was 1:4.1, the age of onset ranged from 20 years to 78 years old, and the median age was 48 years. Patients were divided into three groups according to the pathological subtype. The number of patients belonging to Groups 1, 2, and 3 among the 34 recurrent patients were 11, 20, and 3, respectively. The number of non-recurrent patients belonging to Groups 1, 2, and 3 were 17, 134, and 130 (Table 2), respectively. Among the 34 recurrent patients, 16 patients were treated using surgeries of the first type, 14 patients were treated using surgeries of the second type, and 4 patients were treated using surgeries of the third type. The numbers of non-recurrent patients treated by the first, second, and third types of surgery were 62, 137, and 82, respectively.

**Table 2:** Pathological subtypes of PTC

Pathological subtype	Recurrent group	Non-recurrent group
ConventionalPTC	20	121
Oncocytic variant	7	11
Clear cell variant	0	4
Follicular variant	0	9
Tall cell variant	1	0
Columnar cell variant	3	4
Diffuse sclerosing variant	0	2
Papillary microcarcinoma	3	130

### Univariate analysis of factors related to PTC recurrence

Tumor size, extrathyroid invasion, initial surgery approach, lymph node metastases, and pathological subtype showed statistical differences between the recurrent and non-recurrent groups (*P* < 0.05, Table 1). Larger tumors showed significantly increased risk of recurrence. The recurrence rate of micro-PTC was 2.3 % (3/133) and reached 31.0 % (18/58) when the tumor diameter grew to ≥2 cm. The higher recurrence rates, reaching 35.3 % (12/34), were observed when extrathyroid invasion was present at the time of initial surgery. Initial surgery approach also showed a significant effect on PTC recurrence; recurrence rates could reach 20.5 % (16/78) in patients without lymph node dissection but only 4.7 % (4/86) in patients receiving radical dissection and modified dissection. The recurrence rate was 17.3 % (23/133) in patients with cervical lymph node metastasis at the time of initial surgery and only 6.0 % (11/182) in patients without metastasis. In this research, patients with the tall cell, columnar cell, and oncocytic variants showed higher recurrence rates than patients with other cell variants (Table 2). Factors such as age, gender, complication with HT, and number of lesions demonstrated no correlation with tumor recurrence (*P* > 0.05, Table 1).

### Multivariate analysis of factors related to PTC recurrence

Multivariate analysis revealed that initial surgery approach and pathological subtype were the main factors related to PTC recurrence (*P* < 0.001, Table 3). Initial surgery approach was negatively correlated with PTC recurrence (*β* = −0.320, OR = 0.726). Thus, patients receiving conservative treatment are more likely to develop PTC recurrence than patients treated with more radical modalities. Recurrence rates decreased with increasing surgical scope. Pathological subtype also showed a close correlation with PTC recurrence (*β* = 0.923, OR = 2.517).

**Table 3:** Multivariate analysis of factors related to recurrent PTC

Clinicopathological characteristic	*β*	*SE*	Wald*χ* ^*2*^	*P*	OR	95 % *CI*
Initial operative approach	−0.320	0.093	22.593	<0.001	0.726	0.605 ~ 0.871
Pathological subtype	0.923	0.196	18.337	<0.001	2.517	1.714 ~ 3.696

## Discussion

PTC is the most common but least aggressive histological subtype of thyroid cancer. Most patients with PTC have excellent prognosis. However, recent studies have demonstrated increasing incidence of recurrent PTC [2, 3]. Many factors can affect thyroid cancer recurrence, but final conclusions have not been reached. The results of some studies show that the pathological type, staging, degree of extrathyroid invasion, lymph node metastatic rate, age, and initial surgery approach are related to thyroid cancer recurrence [4, 5]. Our research showed that tumor size, extrathyroid invasion, initial surgery approach, lymph node metastasis, and pathological subtype demonstrate statistically significant differences between the recurrent and non-recurrent groups. By contrast, factors such as age, gender, complication with HT, and number of lesions are not correlated with tumor recurrence. Multivariate analysis results further revealed that initial surgery approach and pathological subtype are main factors related to PTC recurrence.

The dissection methods of primary lesions of PTC include (1) hemithyroidectomy with or without isthmectomy, (2) total/near-total thyroidectomy, and (3) extension of surgical scope. In case of severe extrathyroid invasion, such as invasion of the esophagus, trachea, and nerves, extending the scope of surgery is required. The methods of neck dissection are as follows: (1) central compartment node dissection (unilateral or bilateral), (2) selective neck dissection, (3) functional compartmental en-bloc neck dissection, (4) modified neck dissection, and (5) radical neck dissection [6]. Mazzaferri et al. [7] found that the recurrence rate after partial thyroidectomy is nearly twice that of total and near-total thyroidectomy. By contrast, Cunningham et al. [8] revealed that the recurrence rates had no significant difference between hemithyroidectomy and total/near-total thyroidectomy groups. Monacelli et al. [9] suggested that total thyroidectomy combined with central node dissection must be performed even in the absence of risk factors and without clinically evident nodes. However, some researchers do not advocate prophylactic central neck lymphadenectomy [10]. Univariate analysis in this research showed that initial surgery approach exerts a great impact on the prognosis. For example, while 47.1 % (16/34) of the recurrent PTC patients received the first type of surgery as initial surgery, only 22.1 % (62/281) of the non-recurrent PTC patients received the first type of surgery. About 52.9 % (18/34) of the recurrent PTC patients received the second and third types of surgery. In comparison, 77.9 % (219/281) of the non-recurrent PTC patients received the second and third types of surgery as the initial surgery. Multivariate analysis revealed that initial surgery approach is the main factor related to PTC recurrence (*P* < 0.001); specifically, initial surgery approach demonstrated a negative correlation with PTC recurrence (*β* = −0.320, OR = 0.726).

The recurrence rate decreased with increasing surgical scope. Non-standardized surgical approaches with inappropriately small surgical scopes could lead to tumor residue. Moreover, lesions of lymph node metastasis may be missed, thereby increasing the risk of recurrence. Possible reasons behind the inconsistency of results are as follows: (1) Differences among recruited patients. Patients receiving neck dissection showed significant metastases, whereas no cervical lymph node metastasis was discovered before the operation in patients who did not receive neck dissection. (2) Insufficient number of recruited patients. (3) Difference in surgical techniques among surgeons. Considering these factors, blindly extending or narrowing the surgical scope is irrational.

The World Health Organization (WHO) histological classification of tumors has redefined the subtypes of non-conventional PTC [11]: follicular variant, oncocytic variant, diffuse sclerosing variant, tall cell variant, columnar cell variant, solid variant, PTC with nodular fasciitis-like stroma, clear cell variant, and diffuse follicular variant. PTC patients of different histological subtypes may exhibit diverse clinical and biological behaviors. Subtypes including the tall cell, columnar cell, diffuse sclerosing, and oncocytic variants have higher invasiveness and may promote higher risks of recurrence and metastases [12–14]. The prognosis of patients with the follicular and clear cell variants is similar to that of patients with conventional papillary carcinoma [15]. Thyroid microcarcinoma is a type of papillary carcinoma that is less than 1 cm in diameter with relatively low invasiveness and good prognosis [16]. This research classified histological variants according to the WHO histological classification of tumors. Patients with the tall cell, columnar cell, diffuse sclerosing, and oncocytic variants were classified into Group 1; patients with the follicular, clear cell, and conventional PTC variants were classified into Group 2; and patients with papillary microcarcinomas were classified into Group 3. The research conducted by Boone et al. [17] showed that the recurrence rate of patients with differentiated thyroid carcinoma is lower than that of patients with other types of thyroid carcinoma. Among the differentiated thyroid carcinomas, the recurrence rate of PTC is lower than that of the follicular variant. However, 30 % of the PTC patients continue to suffer from recurrence, metastasis, and even death [18]. Univariate analysis demonstrated that pathological subtype is obviously correlated with PTC recurrence (*P* < 0.01). Multivariate analysis also indicated that pathological subtype is closely related to PTC recurrence (*β* = 0.923, OR = 2.517). The recurrence rate of PTC increased as the invasiveness of the tumor increased. Thus, close follow-up must be carried out in patients with the tall cell, columnar cell, diffuse sclerosing, and oncocytic variants. Effective treatment measures must be taken once recurrence is discovered.

Various results are reported in the literature regarding the effect of lymph node metastases on PTC recurrence. Some studies indicate that lymph node metastases do not affect PTC recurrence [19]. However, some researchers have found that the number of lymph node metastases is associated with postoperative recurrence or re-metastasis. Thus, lymph node metastasis has become an important factor affecting the prognosis and recurrence of thyroid carcinoma [20]. The results of this research revealed statistically significant differences in lymph node metastases between the recurrent and non-recurrent groups. Patients with lymph node metastases at the time of initial surgery are more likely to suffer recurrence than those without metastases. The correlation of cervical lymph node metastases with recurrence needs to be confirmed through large-sample and long-term studies.

Of the 34 recurrent PTC patients in the group, postoperative recurrence intervals ranged from five months to 18 years, with a median time of 46 months. Recurrence was observed to occur within a short period of time. The majority of PTC patients, for example, showed recurrence within two to five years from surgery. Furthermore, recurrence may also occur more than once. Therefore, PTC patients must have regular reexamination with frequent follow-ups within five years after the first treatment. Ultrasound examination must be performed at least once a year within five years after the first treatment for timely discovery of tumor recurrence.

## Conclusions

In conclusion, initial surgery approach, tumor size, extrathyroid invasion, lymph node metastases, and pathological type may be related to PTC recurrence. However, opinions on the resection scope of PTC differ. Surgical indications and methods vary on a case-to-case basis. According to Untch et al. [21], lobectomy for patients with low risk is suitable. Nixon et al. [22] demonstrated no difference in the 10-year survival rate of patients with low-risk highly differentiated thyroid carcinoma and those receiving lobectomy and total thyroidectomy. Doctors must consider patient compliance during follow-ups when evaluating whether or not the patient must receive thyroid lobectomy. For individuals with poor economic means, low education levels, poor knowledge about their condition, and inability to accomplish regular follow-up, complete thyroidectomy may be a suitable option. The most appropriate resection scope for PTC can be determined by considering the thoroughness of operation, the functions of the thyroid and parathyroid glands, and the relationships between important anatomical positions. Enhanced follow-up and postoperative reexamination must be carried out in patients with increased risk of recurrence. Effective and regular treatments must be administered once recurrence is discovered.

## Consent

Written informed consent was obtained from the patient for the publication of this report and any accompanying images.
